# Amino Acid Biosynthesis Inhibitors in Tuberculosis Drug Discovery

**DOI:** 10.3390/pharmaceutics16060725

**Published:** 2024-05-28

**Authors:** Michela Guida, Chiara Tammaro, Miriana Quaranta, Benedetta Salvucci, Mariangela Biava, Giovanna Poce, Sara Consalvi

**Affiliations:** Department of Chemistry and Technologies of Drug, Sapienza University of Rome, Piazzale A. Moro, 5, 00185 Rome, Italy; michela.guida@uniroma1.it (M.G.); chiara.tammaro@uniroma1.it (C.T.); miriana.quaranta@uniroma1.it (M.Q.); benedetta.salvucci@uniroma1.it (B.S.); mariangela.biava@uniroma1.it (M.B.)

**Keywords:** tuberculosis, *Mycobacterium tuberculosis*, amino acids, drug discovery, tryptophan

## Abstract

According to the latest World Health Organization (WHO) report, an estimated 10.6 million people were diagnosed with tuberculosis (TB) in 2022, and 1.30 million died. A major concern is the emergence of multi-drug-resistant (MDR) and extensively drug-resistant (XDR) strains, fueled by the length of anti-TB treatment and HIV comorbidity. Innovative anti-TB agents acting with new modes of action are the only solution to counteract the spread of resistant infections. To escape starvation and survive inside macrophages, *Mtb* has evolved to become independent of the host by synthesizing its own amino acids. Therefore, targeting amino acid biosynthesis could subvert the ability of the mycobacterium to evade the host immune system, providing innovative avenues for drug discovery. The aim of this review is to give an overview of the most recent progress in the discovery of amino acid biosynthesis inhibitors. Among the hits discovered over the past five years, tryptophan (Trp) inhibitors stand out as the most advanced and have significantly contributed to demonstrating the feasibility of this approach for future TB drug discovery. Future efforts should be directed at prioritizing the chemical optimization of these hits to enrich the TB drug pipeline with high-quality leads.

## 1. Introduction

With an estimated 10.6 million people that fell ill and 1.30 million deaths in 2022, tuberculosis (TB), caused by *Mycobacterium tuberculosis* (*Mtb*), remains the world’s second leading cause of death from a single infectious agent after COVID-19 [1]. Despite significant progress being made over the past ten years, global targets remain off track, and urgent action must be taken to reach the goal adopted by the United Nations (UN) and the World Health Organization (WHO) to end the TB epidemic by 2030. A major concern is the emergence of multi-drug-resistant (MDR) and extensively drug-resistant (XDR) strains; according to the most recent WHO report, in 2022, an estimated 410,000 people developed MDR or rifampicin-resistant (RR) TB. As with other bacterial infections, resistance to standard anti-TB drugs has high economic and social impacts and poses a serious threat to global health. In fact, resistance to commonly used antibiotics, often referred to as a “hidden pandemic”, is a leading cause of death, resulting in increased morbidity, mortality, and healthcare expenses. With the increasing risk of returning to a pre-antibiotic era, the need for novel and effective therapeutic strategies has become more pressing [2]. Over the past decade, intensified research efforts have fueled the TB drug pipeline, offering a positive outlook for the future of TB drug discovery [3]. Despite this progress, drugs that act on novel targets are still unrepresented [4]. Innovative anti-TB agents acting with new modes of action are the only solution to counteract the spread of resistant infections. Encouragingly, a deeper understanding of TB biology has resulted in significant progress in identifying new targets for TB drug discovery. While cell wall biosynthesis has been the major focus for years, metabolic pathways have not received as much attention due to concerns about *Mtb*’s ability to extract amino acids from the host and potentially reverse their functions [4]. In recent years, several studies have highlighted the central role of amino acids for *Mtb* survival [5]. Experiments with auxotrophic strains have been useful models to understand metabolic fluxes and the impact of inhibitors, providing growing evidence that auxotrophy for some amino acids makes *Mtb* less virulent and unable to proliferate in the lungs [6,7,8,9,10,11,12,13]. Indeed, amino acid starvation is a mechanism of innate immunity to limit nutrient availability and eliminate pathogenic microorganisms [6]. To escape starvation and survive inside macrophages, *Mtb* has evolved to become independent from the host by synthesizing its own amino acids. Such an autarkic metabolic lifestyle is an evolutionary advantage and a virulence mechanism, holding great promise for target discovery. Therefore, targeting amino acid biosynthesis could subvert the ability of mycobacterium to evade the host immune system, leading to rapid killing in vitro and in vivo. An argument further reinforcing the interest in amino acid biosynthesis is the absence of human orthologues, suggesting that they could serve as excellent anti-TB targets.

The aim of this review is to give an overview of the most recent progress in the discovery of amino acid biosynthesis inhibitors. We only report updated information on inhibitors with documented in vitro activity with a focus on those published between 2018 and 2023. Minimal inhibitory concentrations (MICs), 50% inhibitory concentrations (IC_50_), and 50% cytotoxic concentrations (CC_50_) were converted to µM to allow for an easier comparison of compounds.

## 2. Inhibitors of Amino Acid Biosynthesis

### 2.1. Inhibitors of Aromatic Amino Acid Biosynthesis

**Figure 1:**
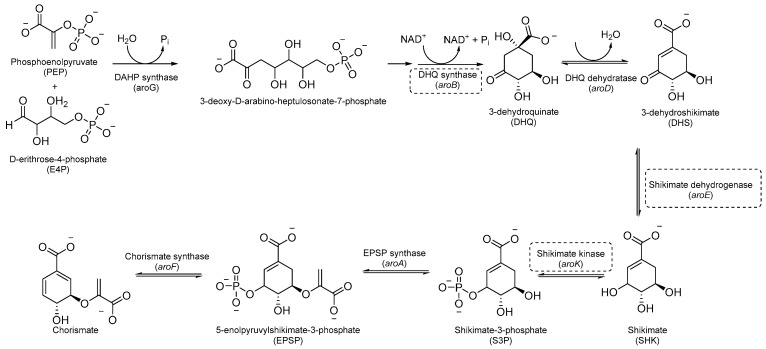
A schematic representation of the shikimate pathway. The most relevant targets are depicted in dotted boxes.

Aromatic amino acids are obtained from carbohydrate precursors through seven enzymatic steps, ultimately resulting in chorismate. This is a metabolic node for phenylalanine (Phe), tyrosine (Tyr), and tryptophan (Trp), as well as for important precursors of *p*-aminobenzoic acid (PABA), *p*-hydroxybenzoate, and isochorismate, which lead to folates, ubiquinone, and mycobactins, respectively [14] (Figure 1). This pathway, also known as the shikimate pathway, is essential for *Mtb* survival [15]. It is only conserved among plants and bacteria and is absent in humans, thus garnering interest for its potential in the development of herbicides and antimicrobials.

The first component of the pathway is 3-deoxy-D-arabino-heptulosonate-7-phosphate (DAHP) synthase. This enzyme catalyzes an aldol condensation between D-erithrose-4-phosphate and phospho-enol-pyruvate (PEP) to produce DAHP and inorganic phosphate, and its vulnerability as a drug target has recently been demonstrated [16]. DAHP is then converted into 3-dehydroquinate (DHQ) by dehydroquinate synthase (DHQS). DHQ dehydratase catalyzes DHQ dehydration into 3-dehydroshikimate, which, in turn, is reduced by shikimate dehydrogenase (SD) into the central metabolite shikimate (Figure 1).

**Figure 2:**
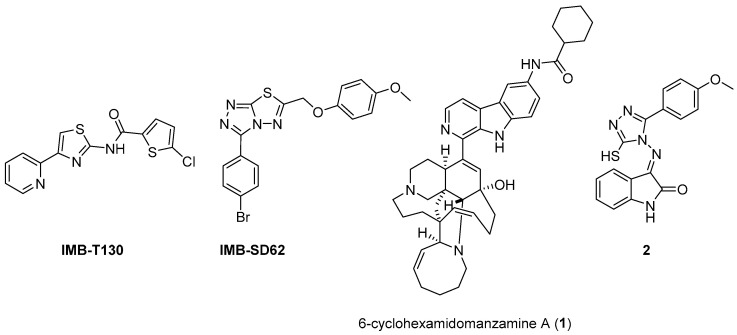
Chemical structures of shikimate pathway inhibitors.

The availability of structural information about the first five enzymes of this pathway has raised the possibility of rational structure-based drug design targeting these enzymes. Even though virtual screenings have generated a growing number of potential inhibitors [17,18,19], there are only a few recent reports about their effective inhibition against *Mtb* [20]. The only two compounds with confirmed activity against *Mtb* are **IMB-T130** and **IMB-SD62** [21] (Figure 2), inhibiting DHQS and SD, respectively. **IMB-T130** (Figure 2) is the identified hit of a phenotypic screening [22] with very good antimycobacterial activity (0.26 µM), low cytotoxicity, and of the ability to inhibit intracellular growth in a dose-dependent manner. Initially, it was speculated that it exerted anti-*Mtb* activity via multitarget activity. Further in vitro studies proved that this compound strongly inhibits DHQS (IC_50_ = 2.86 μM) and has a higher MIC against DHQS-overexpressing strains, suggesting that DHQS could be the target and contribute to its antimycobacterial efficacy. On the other hand, **IMB-SD62** (Figure 2) resulted from the lead optimization of a series of 3,6-disubstituted 1,2,4-triazolo[3,4-b][1,3,4]thiadiazoles identified through a target-based screening against SD [22]. This compound improved the MIC (4.8 µM) and cytotoxicity (CC_50_ = 64.5 µM in Vero cells) compared to its parent compound but showed moderate efficacy in an in vivo model of acute TB infection (1.7 log colony-forming unit (CFU) reduction at 50 mg/kg over a 15-day course of therapy). This could be due to its low solubility and metabolic issues, which limit its absorption and oral bioavailability (14%) and requires further chemical optimization. Moreover, target identification and mechanism of action studies should be carried out to confirm that SD is the direct in vivo target.

The most extensively studied enzyme of this pathway is the shikimate synthase (SK), which catalyzes the fifth step. This protein enables the conversion of shikimate to shikimate-3-phosphate and belongs to the nucleoside monophosphate (NMP) kinase family, which is known for large conformational changes during catalysis. It has an α/β architecture and presents four distinct binding domains: (i) a first NMP binding domain; (ii) a nucleotide binding site; (iii) an LID domain, which is responsible for the binding of Adenosine Triphosphate (ATP); and (iv) a reduced core domain [14]. This was the first enzyme that was proven to be essential for *Mtb* viability [15] and has drawn significant attention over the past 15 years [20]. To date, no SK inhibitors have been tested for their in vivo activity. Indeed, the most relevant studies published in the past five years aimed to identify new chemical entities rather than focusing on the chemical optimization of existing hits. However, different chemical scaffolds, which could be starting points for future optimization studies, have been identified. 6-Cyclohexamidomanzamine A (compound **1**, Figure 2) emerged as the most potent and less toxic inhibitor of 26 marine-derived alkaloids against SK. The marine environment is an important source of biomolecules with a high chemical diversity and a wide range of biological activities [23]. This compound was a mixed non-competitive inhibitor of all forms of SK (free enzyme, enzyme–substrate binary, and tertiary complex). Kinetic profiling predicted a very slow binding time-dependent inhibition mechanism, where compound **1** induces the slow isomerization of the enzyme and the generation of a greater affinity complex. Such a time-dependent component could translate into an enhanced duration of the in vivo effect, which could be a great therapeutic advantage. Therefore, despite the complex structure, this class of compounds is worthy of future investigations, and these alkaloids could serve as novel scaffolds for future chemical optimization [24]. The availability of the crystal structure of SK has facilitated the design of structure-based inhibitors [25,26]. In two independent studies, Dadlani and De Freitas [19,27] adopted a mixed approach by integrating structural data, in vitro testing, and docking simulations. This approach provided a promising compound (MIC = 2.3 µM, SI = 341.63) characterized by a triazole-isatin scaffold (compound **2**, Figure 2). Preliminary structure–activity relationship (SAR) investigations highlighted the importance of the nature, size, and lipophilicity of the substituent on the phenyl ring, paving the way for future research focused on improving the antimycobacterial activities and drug-like properties of this class of molecules.

The last two steps of the shikimate pathway are catalyzed by 5-enolpyruvylshikimate-3-phosphate synthase (EPSP) and chorismate mutase. Even though genetic studies have assessed their vulnerability as drug targets and crystal structures could aid in the design of structure-based inhibitors [28,29], to date, the development of inhibitors is still in its infancy [30,31].

#### Inhibitors of Trp Biosynthesis

**Figure 3:**
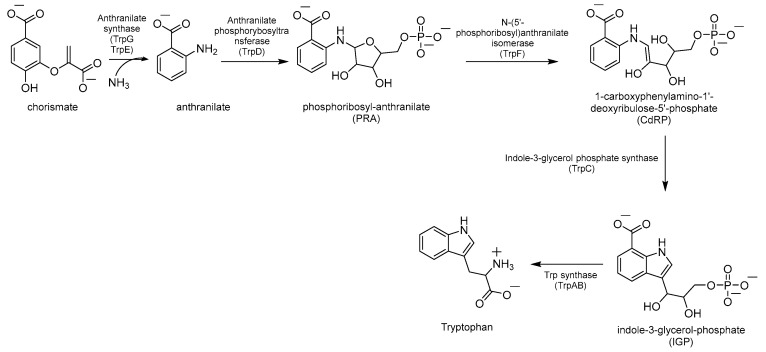
Schematic representation of Trp biosynthesis.

Chorismate is a key metabolic intermediate for Trp biosynthesis. Its conversion to anthranilate is catalyzed by the enzyme anthranilate synthase (AS). AS is a heterodimeric enzyme composed of TrpE (AS-I), which catalyzes the production of anthranilate from chorismate and ammonia, and TrpG (AS-II), a glutamine (Gln) aminotransferase (GAT), which provides the required ammonia by converting Gln to glutamate (Glu) (Figure 3). The anthranilate is then transformed into phosphoribosyl anthranilate (PRA) by the enzyme anthranilate phosphoribosyltransferase (TrpD), which transfers a 5’-phosphoribose unit from phosphoribosyl pyrophosphate (PRPP) onto the amino group of the anthranilate. The ribose ring of PRA is then opened by the isomerase TrpF to give the isomer 1-carboxyphenylamino-1′-deoxyribulose-5′-phosphate (CdRP, Figure 3). This then undergoes a ring closure reaction catalyzed by TrpC, resulting in the indole heterocyclic ring system. The last step is catalyzed by Trp synthase (TrpAB), a bienzymatic complex that exists in tetrameric (αββα) form [32] and is thought to be essential for the survival of *Mtb* in vivo [3]. First, TrpA splits indole-3-glycerol-phosphate (IGP) to form indole and glyceraldehyde-3-phosphate (G3P); then, TrpB condenses indole with *L*-serine (*L*-Ser), forming Trp (Figure 3).

In macrophages, *Mtb* has restricted access to Trp. For this reason, Trp biosynthesis is one of the most investigated and attractive amino acid metabolic pathways for TB drug development [33]. Indeed, Trp auxotrophic strains were less virulent and failed to cause disease in immunocompetent and immunocompromised mice [6]. Trp starvation driven by CD4 T cells is one of the host immune responses after *Mtb* infection [6]. When infected, the macrophage actualizes Trp starvation by expressing indoleamine 2,3-dioxygenase (IDO), an enzyme that catabolizes Trp to kynurenine and other metabolites. IDO catalyzes the first and rate-limiting step of the Trp degradative process, also known as the kynurenine pathway [34,35]. It is one of the most induced genes in both human and mice macrophages infected with *Mtb*. Indeed, Trp and kynurenine levels are extremely different from other metabolites in patients with TB and are highly altered after treatment; in a patient’s plasma, the Trp levels were lower than in those with latent TB and those in the control group and gradually increased after effective TB treatment [36]. This mechanism is successful with other infecting agents that are Trp auxotrophs. On the other hand, Trp starvation can limit *Mtb* growth but fails as a killing mechanism, as *Mtb* is fully competent for its biosynthesis. Moreover, the production of kynurenines, including those generated by IDO, can have immunosuppressive effects: they can inhibit the activation and function of immune cells, leading to immune tolerance, which could allow chronic bacterial infections to persist and evade immune clearance, leading to *Mtb* persisting in a latent state [35]. Inhibitors of the Trp biosynthetic pathway can then function as potential antibiotics by disrupting Trp production in *Mtb* and synergizing with the host immune system to inhibit microbial growth and hinder the establishment and progression of the infection. Additionally, the lack of an equivalent biosynthetic pathway in mammals reduces the likelihood of host toxicity, making these inhibitors even more promising as potential anti-TB agents. This has inspired intense research to identify novel inhibitors of this pathway that could serve as potential antibiotics, many of which have already been reviewed [33].

**Figure 4:**
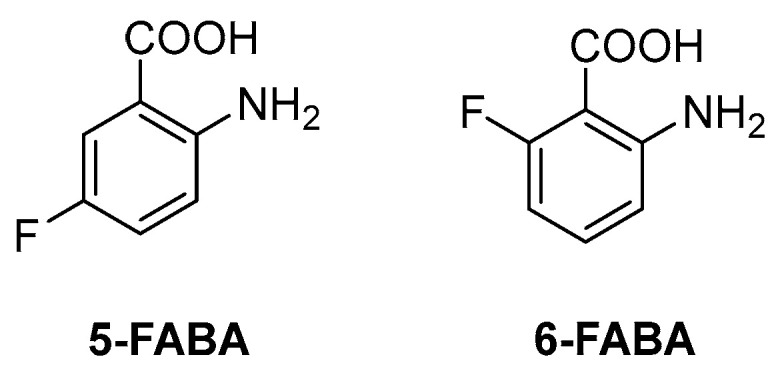
Chemical structures of **5-FABA** and **6-FABA**.

The first inhibitor of this biosynthetic pathway was identified 10 years ago while studying the mycobacterial genetic requirements to endure the CD4 response (the CD4 “counteractome”) [6]. This seminal study demonstrated for the first time that mycobacteria express genes involved in gluconeogenesis and Trp biosynthesis as a response to immune system stimulation in immunocompetent mice. To validate the target, some anthranilate analogues were tested against *Mtb* in the presence and absence of Trp. Two compounds, 2-amino-5-fluorobenzoic acid (**5-FABA**) and 2-amino-6-fluorobenzoic acid (**6-FABA**) (Figure 4), showed an MIC of 5 µM without Trp, while the addition of Trp to the medium rescued *Mtb* growth.

**Figure 5:**
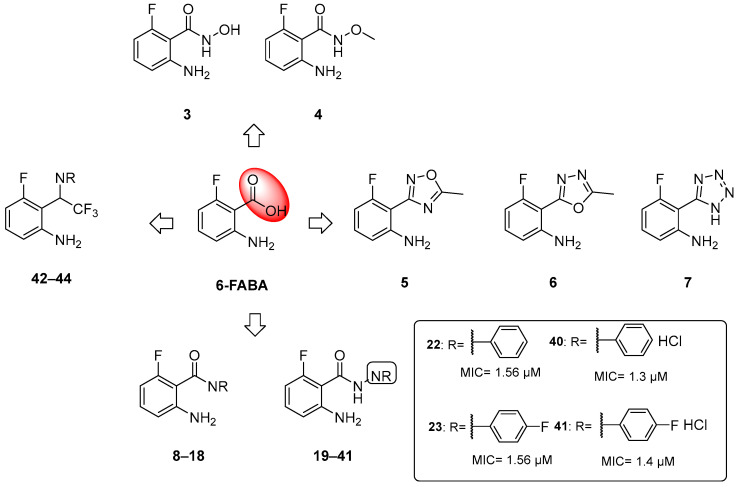
Chemical structures of **6-FABA** analogues and MICs against *Mtb* H37Rv of most active compounds.

When tested in vivo on a murine model of TB infection, the administration of **6-FABA** and its ethyl ester resulted in a significant reduction in the bacterial load in infected mice spleens (10-fold reduction relative to the control) [6], suggesting that the alteration of Trp biosynthesis by an anthranilate-like compound synergizes with the host immune response to *Mtb* infection in vivo. **6-FABA**, the only well-characterized compound, was thought to inhibit either the formation of anthranilate by TrpE or its modification by TrpD. Subsequent work suggested that the toxic mechanism of **6-FABA** occurs downstream, potentially inhibiting a subsequent step or forming fluoro-Trp that is then incorporated into polypeptides, causing global protein stress [37]. Inspired by these findings, our research group developed a class of anthranilate-like compounds (Figure 5) to improve the activity and reduce the cytotoxicity of **6-FABA** [38]. Our strategy was to replace the carboxylic moiety of **6-FABA** with different bioisosters, including hydroxamates (**3**–**4**), oxadiazoles and tetrazoles (**5**–**6**), amides (**7**–**18**), hydrazides (**19**–**39**), aryl hydrazide hydrochlorides (**40**–**41**), and trifluoromethyl amines (**42**–**44**) (Figure 5).

The MICs against *Mtb* H37Rv ranged from 0.625 to >50 µM, with some analogues showing sub-micromolar activities and low cytotoxicities in Vero cells. We observed that the replacement of the carboxylic moiety with a hydrazide (compounds **19**–**41**) led to a significant improvement in both activity and cytotoxicity relative to the parent compound **6-FABA**, showing an MIC between 0.625 and >50 µM. Among them, 18 out of 23 compounds showed an MIC < 9.4 µM in Middlebrook 7H9/DPPC/casitone/Tx. Moreover, the preparation of hydrochloride salts (**40** and **41**) of hydrazides **22** and **23** provided an improvement in activity. Compounds **22**, **23**, and **40** (Figure 5) were tested against bacille Calmette–Guerin (BCG) growth in the presence of Trp and its main biosynthesis precursors, chorismate, anthranilate, and indole, to demonstrate that their growth inhibitory effects were due to their alteration of the Trp biosynthesis pathway. The ability of anthranilate, indole, and Trp to rescue BCG from growth inhibition by these hydrazides suggests that they exert a pleiotropic effect on Trp biosynthesis. By generating **6-FABA**-resistant mutants and evaluating the activity of **40** against these resistors, it was also demonstrated that there is cross-resistance between compound **40** and **6-FABA**, which is in agreement with the speculation that **6-FABA** and hydrazides act by inhibiting the same target. Furthermore, whole genome sequencing (WGS) of resistors against 40 revealed that seven of the nine resistant mutants had mutations in the Trp pathway genes trpE, trpD, trpC, and trpB, confirming that 6-FABA hydrazide analogues exert their anti-mycobacterial activity by interfering with mycobacterial Trp biosynthesis. The lack of a confined mutational spot implies that these mutations modulate the metabolic flux of the biosynthesis pathway rather than directly affecting the binding of compound **40** to a specific enzymatic target. To further investigate the interaction between compound **40** and these genes, we achieved inducible repression by applying CRISPR interference. While the knockdown of *trpD* and *trpB* desensitized BCG to **40**, *trpC* and *trpE* repression had a negligible effect on BCG’s sensitivity. This suggests that these compounds cause functional Trp depletion in mycobacteria by acting as substrate analogues, leading to the production of fluorinated Trp. However, the exact molecular mechanism requires further investigation.

**Figure 6:**
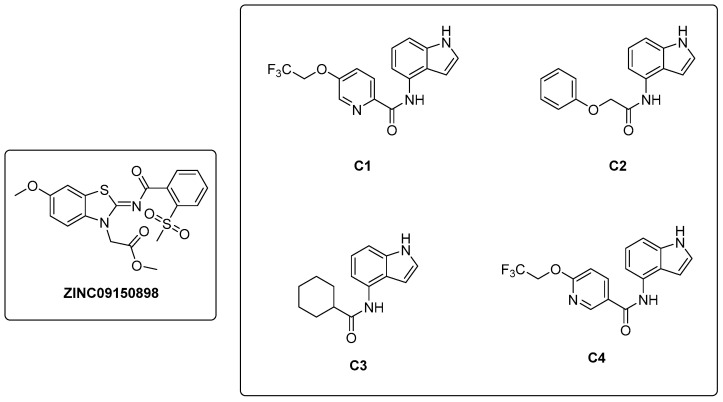
Chemical structures of **ZINC09150898** and indole carboxamides **C1**–**C4**.

In a study conducted by Naz et al. in 2021 [32], a ligand-based pharmacophore modeling approach was employed to identify potent inhibitors against the α-subunit of TrpAB. A virtual screening of drug-like molecules from the ZINC database was performed using a pharmacophore model generated starting from the structures of previously known TrpAB inhibitors and ligands. The best matches were subjected to molecular docking studies against the crystal structure of the α-subunit of TrpAB from *Mtb*. Five hits were then selected on the basis of the best fit value, binding score, binding interaction pattern with catalytically important amino acids in the active pocket, and physicochemical property analysis. These hits were further evaluated for their anti-TB activity through a whole cell-based assay, which led to the identification of a new inhibitor named **ZINC09150898** (Figure 6). It was tested against *Mtb* and showed good antibacterial activity at concentrations up to 27.6 μM, achieving complete growth inhibition (100%) of mycobacteria at 115 μM. To gain insights into the stability of the identified inhibitor in the active pocket of TrpA, molecular dynamics (MD) simulations were performed. Various analyses were conducted to assess the binding mode stability of the inhibitor, whose binding score was determined to be −32.07 kcal/mol. Van der Waals interactions were demonstrated to play a crucial role in the retention of the inhibitor within the protein pocket through a molecular mechanics–Poisson–Boltzmann surface area (MM-PBSA) analysis. The authors also identified Gly69, Ser70, and Asp68 as key residues for the binding by performing site-directed mutagenesis; changing these residues into alanine (Ala) resulted, in fact, in a reduction in the binding affinity of the inhibitor.

In 2021, Libardo et al. [39] identified a series of indole-4-carboxamides with potent antitubercular activity. Representative members of the series (Figure 6) were selected to understand their antimycobacterial properties and how they inhibit the growth of *Mtb*. Compounds **C1**–**C4** exhibited potent activities, inhibiting *Mtb* growth at low mM concentrations. Additionally, compound **C1** was non-toxic to J774 macrophages and showed a reduction in intracellular *Mtb* titer in a murine macrophage model of infection. The mechanism of action was determined through the generation of **C1**-resistant mutants. Interestingly, indole-4-carboxamides act as prodrugs: AmiC-mediated hydrolysis converts them to 4-aminoindole (4-AI), an antimetabolite, which is then metabolically incorporated by Trp synthase to form cytotoxic 4-aminotryptophan. *Mtb* developed resistance to indole 4-carboxamides through three distinct mechanisms: (*i*) a decrease in drug metabolism, which reduces the conversion of the prodrug to the active cytotoxic compound; (*ii*) a specific mutation in *trpE* that makes the enzyme resistant to feedback inhibition, determining an increased biosynthetic flux in Trp that compensates for the cytotoxic effects of 4-aminotryptophan; and (***iii***) an in situ enzymatic attenuation of TrpAB, further reducing the incorporation of 4-AI into Trp biosynthesis, a genuine resistance mechanism in mycobacteria. This study unveils a very complex mechanism of metabolic flux regulation in mycobacteria, which can escape killing by toxic false metabolite flux either through a loss of feedback allosteric regulation, enhancing the flux of intermediates, or through enzymatic attenuation, which reduces the concentration of toxic metabolites. The medicinal chemistry optimization of 4-AI is essential to obtain a suitable candidate for in vivo studies, obtain further insights into its mechanism of action, and overcome resistance [40].

### 2.2. Inhibitors of Branched Chain Aminoacid (BCAA) Biosynthesis

**Figure 7:**
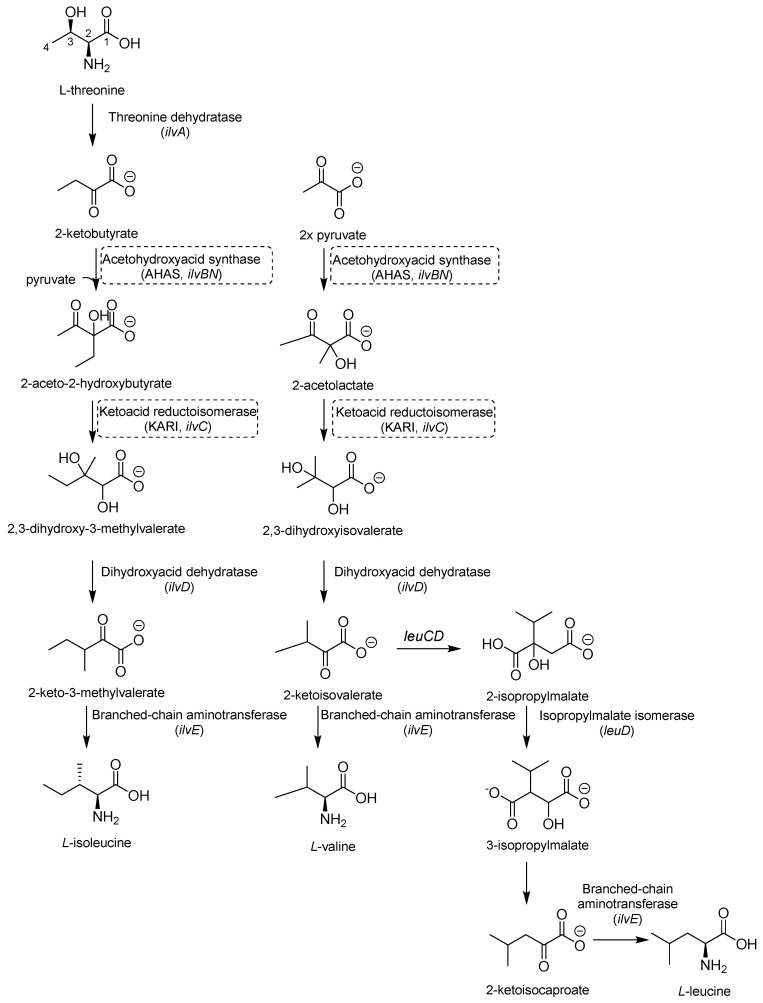
A schematic representation of BCAA biosynthesis. The most relevant targets are depicted in dotted boxes.

Valine (Val), leucine (Leu), and isoleucine (Ile) share the first part of their biosynthesis (Figure 7). This biosynthetic pathway exists in plants, fungi, and bacteria but lacks a homolog in mammals, making the enzymes of this pathway attractive targets for drug development. The first enzyme in the pathway is acetohydroxyacid synthase (AHAS) encoded by *ilvB1* for the largest domain and *ilvN* for the minor subunit. *IlvB1* mutants are auxotrophic, and the resulting depletion of BCAAs leads to *Mtb* death [41]. The second promising target is a ketoacid reductoisomerase (KARI), a bifunctional enzyme that first catalyzes a methyl migration, requiring Mg^2+^ for its activity, and then reduces in the presence of nicotinamide adenine dinucleotide phosphate (NADPH). Finally, the gene *ilvD* encodes for dihydroxyacid dehydratase, which catalyzes one of the middle steps of Val and Ile biosynthesis. The *ilvD* mutant is auxotrophic as well, inspiring the development of inhibitors [7,42].

#### 2.2.1. AHAS Inhibitors

AHAS comprises a catalytic (IlvB1) and a regulatory subunit (IlvN) and catalyzes the condensation of two pyruvate molecules to acetolactate and pyruvate with 2-ketobutyrate to form 2-aceto-2-hydroxybutirate in the second step of BCAA biosynthesis (Figure 7). It is considered a biologically safe target for common herbicides, such as sulfonylurea sulfometuron methyl (SMM) and triazolopyrimidine sulfonamides. Interestingly, high doses of SMM were efficacious in a murine model of TB infection [43], disclosing the therapeutic potential of this target and inspiring the design of several analogues with improved activities [44,45,46].

**Figure 8:**
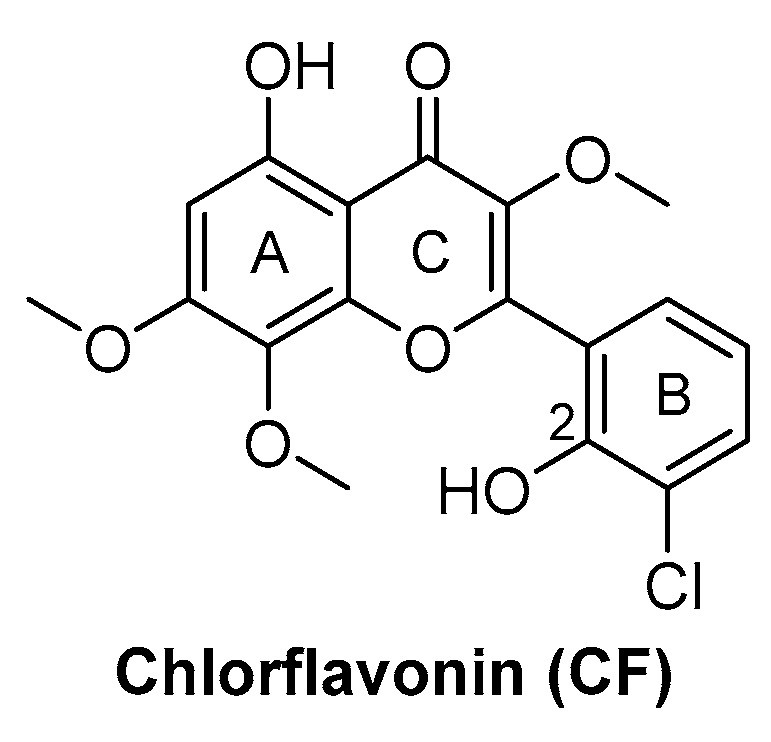
CF chemical structure.

Chlorflavonin (CF) (Figure 8) is an interesting hit of a new class of AHAS inhibitors that are structurally unrelated to the above-mentioned molecules. It was purified from an extract of *Moringa stenopetala* and displayed good antimycobacterial activity (MIC_90_ = 1.56 µM), no cytotoxicity at 100 µM against the two human cell lines THP-1 and MRC-5, a high selectivity index (SI) (IC_50_/MIC_90_ ≥ 64), and intracellular activity in infected macrophages. Mode of action and resistance mechanism experiments revealed that CF inhibits AHAS enzymatic activity by binding the ILvB1 catalytic subunit, causing a combined auxotrophic effect on Val, Ile, Leu, and pantothenic acid. The inhibitory effect is completely reversed through medium supplementation with BCAAs and pantothenic acid, suggesting that there are no relevant off-target effects for antibacterial activity. Docking studies predicted that CF could interact with the catalytic subunit IlvB1 through a hydrogen bond and a salt bridge with Lys 197 and π−π interactions between the phenyl moiety and Trp 516. Chlorine, which seemed to be essential for the activity, pointed toward a subunit pocket defined by Leu 65. Target engagement was confirmed by in vitro inhibition studies with recombinant ILvB1, which confirmed the direct interaction of CF with its target [47]. Recently, Berger and co-workers developed a five-step synthetic route to prepare CF and synthesized a small library of analogues by modifying the B ring of the scaffold (Figure 8). Unfortunately, any change in the B ring led to a loss of activity of the derivatives [48].

#### 2.2.2. KARI Inhibitors

KARI catalyzes the transfer of a methyl group to generate the intermediate 3-hydroxy-3methyl-2-ketobutyrate and its reduction to *R*-2,3-dihydroxyisovalerate in the third step of BCAA biosynthesis (Figure 7). It is a bifunctional enzyme which requires Mg^2+^ for the methylation and NADH or NADPH for the reduction and has recently gained considerable attention as an innovative target in anti-TB drug discovery [49]. Lin and co-workers identified a promising compound (**NSC116565**, K*_i_* = 0.0954 μM, Table 1) through a screening of the National Cancer Institute-Development Therapeutics Program (NCI-DTP) library. Kinetic, calorimetric, and structural studies showed that **NSC116565** is a potent, competitive, and time-dependent inhibitor, which can bind the enzyme both in the presence and absence of NAPDH with K*_D_* values of 2.0 and 2.8 µM, respectively. To confirm that it acts as a KARI inhibitor, its biological activity was investigated with and without BCAA supplementation in the medium culture. In the absence of BCAAs, **NSC116565** was able to inhibit the growth of *Mtb* H37Rv with an MIC_50_ of 6.06 µM and an MIC_90_ of 20.42 µM, while its MIC_90_ was >30 µM upon the addition of 1 mM of BCAA. **NSC116565** did not display cytotoxicity in CD2F1 mice at doses of up to 300 mg/kg, confirming that it is a promising hit for further structural optimization [49,50].

*N*-hydroxy-*N*-isopropyloxamate (**IpOHA**) (Table 1) is a herbicide that inhibits KARI in the nanomolar range. This compound acts as a transition-state analogue and is a time-dependent inhibitor of the enzyme. Twenty-two **IpOHA** analogues showed improved activities against the target, but none of them were active against *Mtb* [51]. This lack of activity was likely due to the hydrophilicity of this class of compounds, which limits their permeability through the cell wall. To improve lipophilicity, five prodrugs (**42 a**–**e**) of the most active analogue were prepared by esterifying the carboxylate group with alcohol functions of different lengths. Esterification with octanol, dodecanol, and decanol generated compounds with MIC_90_ values in the range of 2–3 μM against H37Rv strains. The addition of BCAAs to the media reversed the activity (MIC_90_ > 30 μM), confirming that these compounds act by inhibiting the BCAA pathway [52].

A screening of the Medicines for Malaria Venture Pathogen Box (MMVPB) against *Mtb*-KARI produced a potential hit (**MMV553002**, Table 1), with a K_i_ value of 0.153μM, an MIC between 0.8 and 19 μM, and no cytotoxicity against human HepG2 cells [51]. Kinetic studies revealed that this compound is rapidly hydrolyzed to 3-(methylsulfonyl)-2-oxopropanoic acid (4-EP) and 2-aminophenol (2-AP). Inhibition and crystallographic studies revealed that 4-EP is a time-dependent inhibitor and strongly interacts with Mg^2+^ ions inside the active site of the enzyme, similar to **IpOHA**. However, its charge and hydrophilic character prevent it from crossing the cell wall, and the anti-TB activity of **MMV553002** is only due to its metabolite, the 2-amino-phenol. Four analogues were synthesized, but none of them were more active than the parent compound [51].

In recent years, Krishna et al. identified a new hit (compound **43**, Table 1) acting against *Mtb*-KARI through a virtual screening of an *in-house* database. This thiadiazine derivative was able to inhibit *Mtb*-H37Rv growth with an MIC of 2.06 μM. To improve the inhibitory activity of the hit, 22 analogues were prepared by modifying the thiophen ring with bioisoster furan and by introducing various benzene-substituted rings. The ureidic moiety, the electron withdrawing groups on the thiophen, and the thiadiazine core were left unchanged as they seemed to be essential for the activity. The most promising compound was **44** (Table 1), which also showed encouraging activities in infected macrophages and in a nutrient starvation model. However, further studies are needed to clarify its mechanism of action and to determine whether KARI is the only target [53,54].

**Table 1:** Chemical structures, SAR plan, and most advanced KARI inhibitors.

Hit/Scaffold	SAR Plan from Hit	Most Advanced Analogue	Refs.
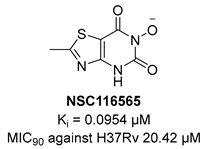	-	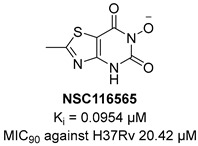	[50]
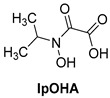	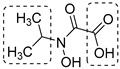	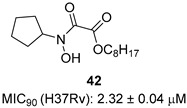	[52]
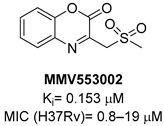	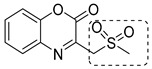	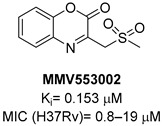	[51]
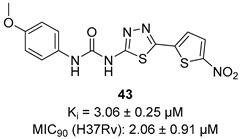	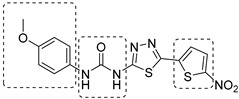	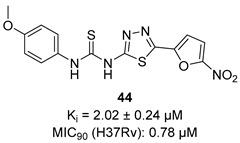	[53,54]

### 2.3. Inhibitors of Arginine (Arg) Biosynthesis

**Figure 9:**
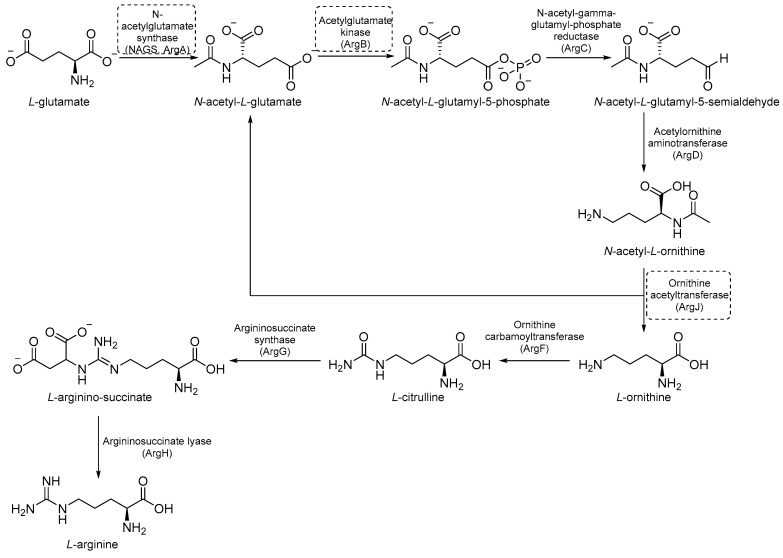
A schematic representation of Arg biosynthesis. The most relevant targets are depicted in dotted boxes.

Arg biosynthesis (Figure 9) consists of eight different steps, each catalyzed by a different enzyme (argA-D, argF-H, and ArgJ) [55]. Like other microorganisms and cancer cells, the upregulation of de novo Arg biosynthesis is also an early response to oxidative stress. Unlike other amino acids, Arg deprivation leads to rapid sterilization. It has been demonstrated that Arg-mediated cell death in *Mtb* is caused by reactive-oxygen species (ROS)-mediated oxidative damage. Indeed, experiments with auxotrophs showed that Arg starvation and the accumulation of upstream metabolites caused a complex metabolic disruption, which ultimately led to rapid sterilization through the depletion of antioxidant thiols and ROS-mediated oxidative damage [8]. Its critical sterilization role and the absence of human homologues for many of the enzymes of Arg biosynthesis make the Arg biosynthetic pathway one of the most attractive targets for anti-TB drug development [55].

#### 2.3.1. ArgA Inhibitors

ArgA is the first enzyme in the Arg biosynthetic pathway and catalyzes the transfer of an acetyl group from acetyl coenzyme A (AcCoA) to *L*-Glu. It is classified as an *N*-acetylglutamate synthase (NAGS) and belongs to the GCN5-related *N*-acetyl transferase superfamily [56]. ArgA is regulated by negative feedback through the binding of *L*-Arg to the active site [57].

**Figure 10:**
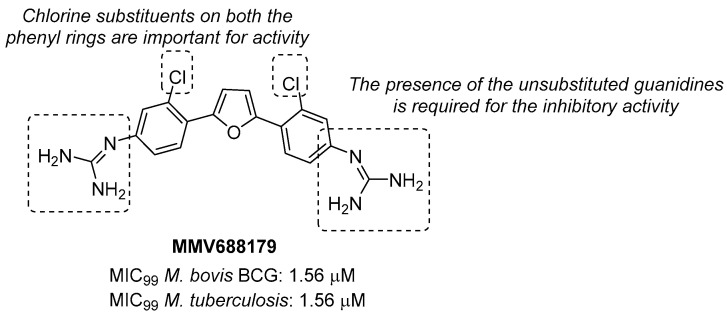
Chemical structure, antimycobacterial activity, and preliminary SAR of **MMV688179**.

Through the screening of the National Institutes of Health (NIH) Diversity set and Pathogen box, Khurana et al. identified a symmetric dicationic 2,5-bis (2-chloro-4-guanidinophenyl) furan (**MMV688179**, Figure 10) that inhibits *Mtb* growth in vitro with an MIC_99_ of 1.56 µM. This hit kills *Mtb* in a dose-dependent manner. As suggested by the MD simulation, it seems to strongly bind the *L*-Arg binding pocket, acting as an allosteric ArgA inhibitor.

The proposed mechanism of action is the cell damage induced by ROS production in Arg starvation conditions, which leads to DNA damage and then cell death. The oral administration of **MMV688179** only slightly inhibited mycobacterial growth after two weeks of treatment, and no antimycobacterial activity was observed in the lungs and spleen at 4 weeks post-infection. This lack of efficacy could be due to poor pharmacokinetic (PK) properties, and new analogues of **MMV688179** were synthesized to expand the SAR of this new class of inhibitors. While shifting the chlorine atom to a different position did not impact the activity, the replacement of the two chlorine atoms with bromines lowered the potency, confirming that chlorine is the most appropriate halogen to guarantee antimycobacterial activity. Moreover, the freedom grades of the terminal guanidine groups seem to be required for essential interactions in the active site; indeed, both substituents on the guanidines and bulky substituents at position 3 on the phenyl rings reduce the activity as they might hamper the interactions between the nitrogen atoms and the active site (Figure 10) [58]. Unfortunately, none of the synthesized compounds were more active than the parent compound.

#### 2.3.2. ArgB Inhibitors

**Figure 11:**
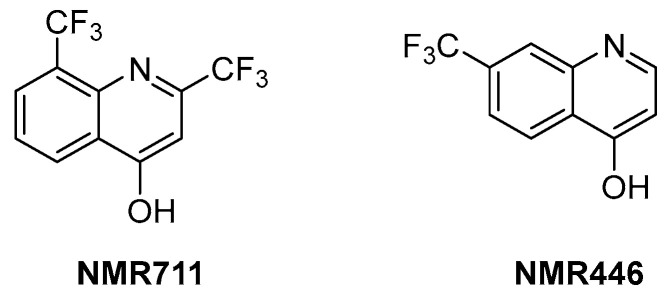
Chemical structures of **NMR711** and **NMR446** fragments.

ArgB is an acetylglutamate kinase that catalyzes the second step of Arg biosynthesis (Figure 9). This enzyme holds great potential to be a good target for the development of new antitubercular drugs because it lacks a homologue in humans and is essential for *Mtb* growth and survival [8]. Still, it remains largely unexplored. In 2021, its ligandability was explored through a fragment-based approach. The screening of a library of 960 fragments yielded two structurally related positive hits: **NMR711** and **NMR446** (Figure 11). Crystallographic data demonstrate that both fragments tightly bind to an interfacial allosteric site in ArgB through π–π interactions and few hydrogen bonds, with both trifluoromethyl moieties occupying the same position in the enzyme pocket. **NMR711** and **NMR446** have IC_50_ values of 366 and 707 µM, respectively, against ArgB. Isothermal Titration Calorimetry (ITC) experiments confirmed the binding (K*_D_* = 7.7 and 23 µM, respectively), and Nuclear Magnetic Resonance (NMR) competition assays clarified that these derivatives do not compete with other ArgB natural ligands such as ATP, *N*-acetyl glutamate (NAG), and *L*-Arg. The MICs against *Mtb* H37Rv, Δ*arg*B-c, and the MDR strain V2475 were 90–180 µM and 117–234 µM for **NMR711** and >200 µM and >200 µM for **NMR446**. However, only **NMR446** was inactive after adding *L*-Arg to the media, proving to be on-target. This study offers a proof of concept of the potential of this target and provides two interesting fragment hits for further fragment growth and medicinal chemistry campaigns. However, the intrinsic hydrophobic nature of the interfacial binding site of ArgB, as well as the lack of hydrogen bonds and polar contacts, strongly limits fragment development and drug discovery programs, and the potential of this class of inhibitors remains largely unexplored [59].

#### 2.3.3. ArgJ Inhibitors

**Figure 12:**
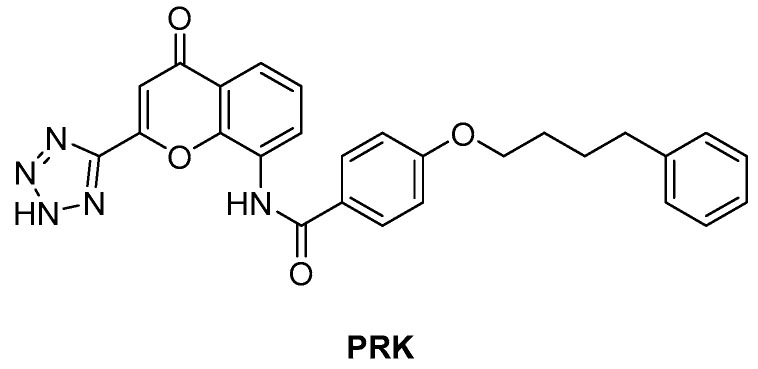
Chemical structure of **PRK**.

*ArgJ* encodes for an ornithine acetyltransferase (OAT) involved in the acetyl recycling in Arg biosynthesis and catalyzes the transfer of an acetyl group from *N*-acetylornithine (NAORN) to *L*-Glu to form *N*-acetyl *L*-Glu (Figure 9) [60]. This attractive target lacks a homologue in humans and is essential for mycobacterial survival [61]. However, its druggability has remained unexplored for years. A possible explanation could be that developing substrate analogue inhibitors could be accompanied by off-target effects since many substrates of the Arg pathway are common to host cellular pathways. To avoid off-target effects and explore the potential of this target, Mishra and coworkers rationalized an inhibitor of the allosteric site, which is less evolutionary conserved and should ensure selectivity. To avoid safety issues, a library of 1556 Food and Drug Administration (FDA)-approved drugs was selected for virtual screening against ArgJ. To provide a proper characterization of ArgJ for drug targeting, this study combined in silico and extensive biochemical studies as well as functional in vitro strategies. The most promising hit of this screening was Pranlukast (**PRK**, Figure 12), an antagonist of the cysteinyl leukotriene receptor that is effective for the treatment of asthma. This drug acted as an allosteric modulator by binding to a novel pocket in *Mtb* ArgJ, inhibiting the activity in a non-competitive manner. It markedly inhibited the growth of *Mtb* H37Rv (MIC_90_ = 10 µM) and was also effective against *Mtb*-infected monocytic THP-1 cells without detrimental effects on host cell survival. Moreover, it reduced *Mtb*-mediated apoptosis in macrophages and showed a synergistic effect with standard-of-care anti-TB drugs. This drug has a dual mechanism of action: Firstly, the Arg biosynthesis inhibition mediates the *Mtb* killing mechanism, as confirmed by Arg supplementation experiments. Secondly, it enables the efficient intra-macrophage elimination of mycobacteria by targeting the 5-lipooxigenase pathway, which facilitates *Mtb* survival and growth in host macrophages. Encouragingly, **PRK** also induced a significant decrease in the granuloma size in a chronic murine model of TB infection and showed a remarkable additive effect in combination with rifampicin (RIF), confirming its potential in combination therapies [41,62]. Moreover, PK studies revealed that the plasma concentration after single and multiple doses achieves levels close to the IC_50_ range, suggesting a translational value [63].

### 2.4. Inhibitors of Gln Biosynthesis

**Figure 13:**
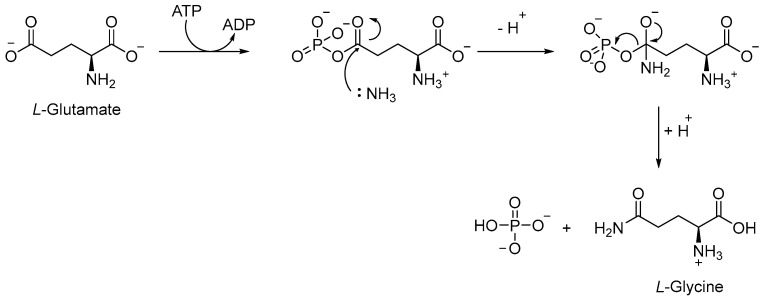
Conversion of *L*-Glu to *L*-Gln, catalyzed by GlnA1.

The Gln biosynthetic pathway is essential for both the production of *L*-Gln and *L*-Glu and nitrogen metabolism. Moreover, it is implicated in host defense against TB, serving as a carbon and nitrogen source for M1 macrophage polarization. Gln synthetase (GS or GlnA1) is an ATP-dependent enzyme that catalyzes [64] the reaction between the ammonium ion and *L*-Glu to form *L*-Gln, Adenosine Diphosphate (ADP), phosphate, and *L*-Glu (Figure 13). *Mtb* has four different GS genes, but only *GlnA1* is essential for both in vitro and in vivo survival [65]. The main advantage of targeting GlnA1 is that its metabolites are involved in the formation of a poly-*L*-Glu/Gln structure, a constituent of the cell wall of mycobacteria [66,67]. Thus, the inhibition of this enzyme can affect both amino acid biosynthesis and the integrity of the cell wall, leading to the death of mycobacteria [68,69,70]. GlnA1 is a dodecameric enzyme, and the active site is formed by two cones connected at their narrow ends. When ATP binds to the enzyme, in the presence of two or three metal ions (Mg^2+^ or Mn^2+^), it passes from a relaxed state to a *taut* (active) state, also known as closed conformation [66,69]. In general, the mechanism allows for the entry of *L*-Glu and ammonium ion from one side, while ATP passes via the opposite side of the cone. The first step is the phosphorylation of *L*-Glu, followed by the nucleophilic attack of the ammonium ion to generate the amide moiety of *L*-Gln (Figure 13).

**Figure 14:**
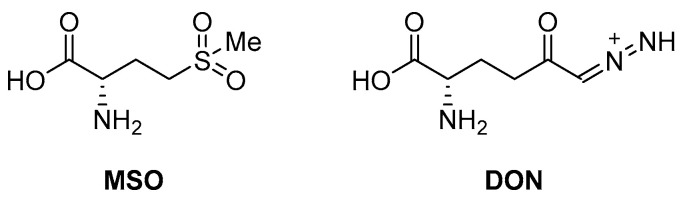
Chemical structures of *L*-Gln antagonists **MSO** and 6-diazo-5-oxo-l-norleucine (**DON**).

**Figure 15:**
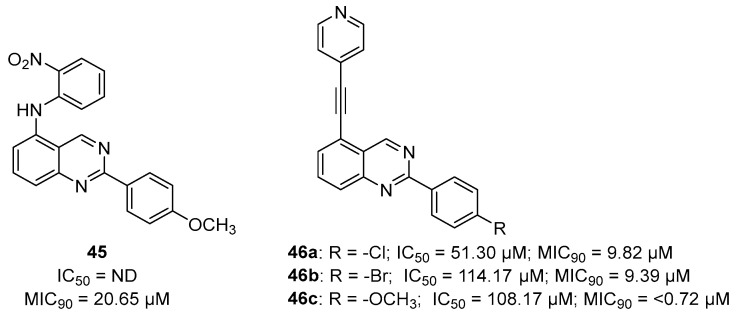
Chemical structures and antimycobacterial activities of 4-pyridylamino and 4-(ethynylpyridine) quinazolines against *Mtb* H37Rv.

An early seminal study demonstrated the druggability of this target by showing that the known epileptogenic agent, methionine sulfoximine (**MSO**) (Figure 14), which possesses direct antibacterial activity on a solid medium (MIC = 50 µM), can reduce the bacterial burden in a guinea pig model, and it protects animals from weight loss and has synergistic activity with isoniazid (INH) in vivo. However, the high frequency of spontaneous resistant mutants to **MSO** has dampened the enthusiasm in developing analogues against this target. Additionally, the amino acid binding site in GS exhibits a structure that is highly conserved in humans. For this reason, the ATP binding site has become a more attractive site for targeting for the development of new anti-TB drugs with reduced off-target effects [71,72,73]. Most recently, Dilebo et al. optimized a novel series of 4-pyridylamino and 4-(ethynylpyridine) quinazolines (Figure 15). All of these derivatives were subjected to in vitro anti-*Mtb* assays, cytotoxicity studies, and docking studies to predict their binding modes to the target enzyme. The 4-(ethynylpyridine) quinazolines’ MIC_90_ values were generally low (0.72 µM < MIC_90_ < 23 µM). The best activity was related to the presence of a methoxyl, which can probably engage hydrogen bonds with the target. The best compound of this series showed a very good SI and could serve as a useful starting point for further chemical optimization to expand this series. However, further metabolomic experiments and genetic and biochemical validation are needed to confirm the target [74].

**Figure 16:**
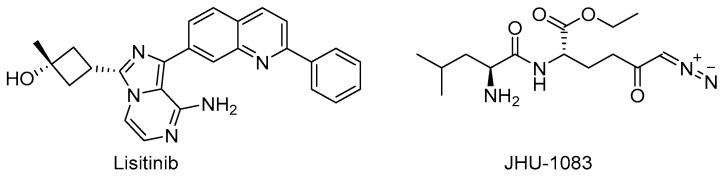
Chemical structures of **LIN** and **JHU-1083**.

Target-based high-throughput screening (HTS) of the MMVPB against GlnA1 revealed that Lisitinib (**LIN**, Figure 16), a clinical-stage kinase inhibitor targeting insulin-like growth factor 1 and insulin receptor (IGF1R/IR), is a potent GlnA1 inhibitor (IC_50_ = 1 µM). Kinetic enzyme assays showed that **LIN** acts as an ATP-competitive inhibitor, and it was hypothesized that this mechanism of action is due to the imidazopyrazine moiety, which mimics the adenine of the natural ligand ATP quite well and establishes π-π interactions with the benzene of Phe232 of GlnA1. GlnA1 inhibition results in weak direct antimycobacterial activity, probably due to permeability issues, which require further chemical optimization. However, an analysis of the *Mtb*-infected THP-1 macrophages cell responses showed that **LIN** treatment enabled the intracellular killing of drug-sensitive and -resistant *Mtb* in a dose-dependent manner through autophagy activation [75]. Moreover, Phase I studies showed that it has excellent properties in terms of PK and metabolism, achieving plasma concentrations that are well correlated to the IC_50_ value [76]. Therefore, this early hit is worthy of chemical optimization to overcome permeability issues, increase direct antimycobacterial activity, and develop a novel class of anti-TB host-directed therapy (HDT) dual targeting inhibitors [75]. Even though GlnA1 has been extensively studied as a direct anti-TB drug target [68,69,77,78], HDT is the most emerging role in Gln metabolism inhibition. It is well-known that Gln metabolism antagonism is also a strategy to improve T-cell immunity effectors in a tumor microenvironment, but the mechanism in *Mtb* has only been disclosed recently. **JHU083** (Figure 16), a prodrug developed to improve the therapeutic index of the Gln antagonist **DON** (Figure 14), has promising anticancer activity in preclinical models and acts by enhancing T-cell activity through metabolic reprogramming. A very recent study demonstrated that **JHU-083** has a dual antibacterial and host-directed effect against TB [79]. Despite showing modest direct anti-TB activity, **JHU-1083** treatment significantly reduced TB infection in murine models (1.9 log_10_ bacteria reduction after 5 weeks of treatment), leading to an improved anti-*Mtb* immune response in all treated animals. Its therapeutic efficacy requires an intact immune system and was completely abrogated in immunocompromised mice, supporting the hypothesis that the immunomodulatory effect, more than the direct antibacterial effect, was the predominant mechanism of its therapeutic efficacy. Moreover, the metabolic fluxes of the immunologically relevant amino acids Trp and Arg are altered following **JHU-1083** treatment, which is consistent with an enhanced host immune response. This study also sheds light on an additional role of GlnA1, which could be exploited as a virulence factor to increase Gln metabolism in the granuloma, leading to an immunosuppressive environment (reduced T-cell function, decreased citrulline and NO production, and immunosuppressive myeloid accumulation), which promotes disease progression.

### 2.5. Inhibitors of Ser Biosynthesis

**Figure 17:**
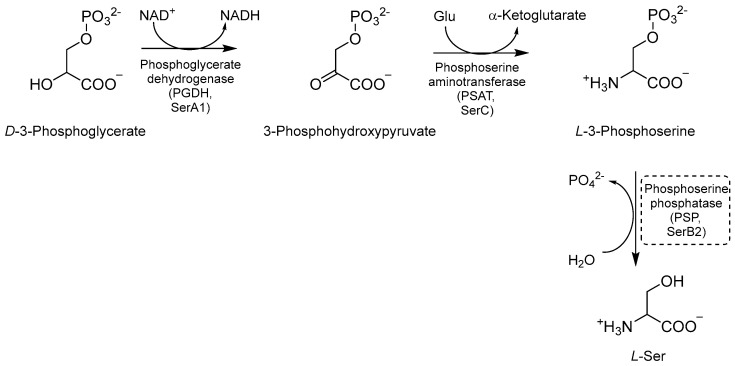
A schematic representation of Ser biosynthesis. The most relevant target is shown in a dotted box.

Ser biosynthesis is a crucial metabolic pathway that enables *Mtb* to synthesize *L*-Ser. This amino acid is required for *Mtb* growth and survival, and it is involved in various cellular processes such as nucleotide synthesis, redox balance, and cell wall biosynthesis [36,80,81]. The first step of this pathway is the NAD^+^-mediated oxidation of *D*-3-phosphoglycerate to 3-phosphohydroxypyruvate, catalyzed by the enzyme phosphoglycerate dehydrogenase (PGDH or SerA1) [81,82] (Figure 17). Next, phosphoserine aminotransferase (PSAT or SerC) transforms phosphohydroxypyruvate into *L*-3 phosphoserine using glutamate as an amino donor [80,81]. Finally, L-3 phosphoserine undergoes a dephosphorylation step mediated by the enzyme phosphoserine phosphatase (PSP or SerB2), leading to the formation of *L*-Ser. This step is essential for generating free Ser molecules that can be exploited by mycobacteria for various metabolic processes [81,82]. *L*-Ser is also a substrate for the biosynthesis of other important metabolites, such as glycine (Gly), an important precursor for the synthesis of nucleotides and peptidoglycan, and phosphatidylserine (PS), a major component of the bacterial cell membrane contributing to the maintenance and integrity of the bacterial cell envelope. Therefore, gaining a clear understanding of the intricacies of this pathway may provide insights into many potential targets for the development of novel therapeutic strategies against tuberculosis [36].

Even though these three enzymes are essential for *Mtb* growth and survival [61], no inhibitors of SerA and SerC have been reported so far, and only a few inhibitors on SerB have been developed over the past five years. Indeed, the development of compounds targeting this pathway is limited by the existence of human orthologues, which share very similar catalytic pockets. Moreover, the lack of a thorough structural characterization hinders the structure-based design and the drug discovery process.

*Mtb*-SerB2 is predominantly secreted in the cytosol of infected macrophages (specifically in THP-1 cells), where it triggers a rearrangement of the cytoskeleton, creating a necrotic environment. These conditions promote the growth of *Mtb* and contribute to intra-macrophagic survival. *Mtb*-SerB2 is then considered an invasive secreted virulence factor employed by *Mtb* to evade the host immune system. Its phosphatase activity can alter the immune response by interacting with cofilin, nuclear factor-kappa B (NFkB), and P38 and by inhibiting the expression of interleukin 8 (IL-8), an important immune mediator [81,83]. In terms of structure, SerB2 possesses a core domain similar to the Rossman fold, and it is composed of α and β elements. This core domain comprises four loops that encompass all three motifs that are essential for PSP activity. Additionally, SerB2 includes a cap domain that acts as a closure for the active site, enabling specific recognition of the substrate. The catalytic core of SerB2 exhibits the characteristic features that are commonly observed in HAD (haloacid dehalogenase) phosphatases [83]. *Mtb*-SerB2 possesses two regulatory N-terminal Aspartate kinase, Chorismate mutase, and TyrA (ACT) amino acid-binding domains. These domains serve as conserved allosteric regions responsible for the regulation of enzymatic activity when exposed to high levels of reaction products. Notably, human PSP only comprises the phosphatase domain, making the ACT domains potential targets for the development of small inhibitors that could selectively inhibit *Mtb*-SerB2 [81]. Most of the known inhibitors of SerB2 target the catalytic binding site and were identified through the target-based screening of commercial libraries [84,85] (Table 2).

**Table 2:** Chemical structures, SAR plan, and most advanced SerB inhibitors.

Hit/Scaffold	SAR Plan from Hit	Most Advanced Analogue	Refs.
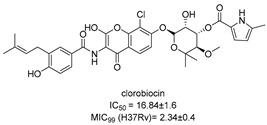	-	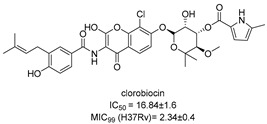	[84]
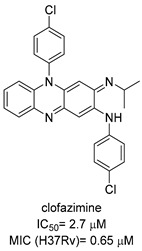	-	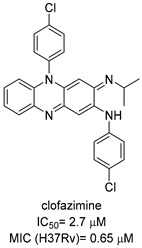	[85]
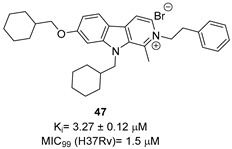	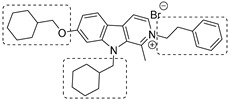	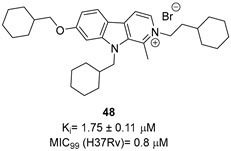	[86]

To increase chemical diversity, Pierson et al. [86] tested an in-house chemical library with a great range of novel chemical scaffolds. Interestingly, three hits were harmine-derived with bulky aliphatic or aromatic substituents and shared a 2,7,9-trisubstituted pattern, indicating the crucial involvement of the positive charge carried by the N2 atom on their activity. Compound **48** (Table 2) was synthesized to investigate the importance of aromatic substituents and was found to be the most potent. This confirmed the importance of flexible and hydrophobic substituents and suggested that aromatic residues are not involved in interactions within the binding site. Indeed, the three substituents on the β-carboline core occupy distinct pockets within the active site, primarily composed of hydrophobic residues such as Leu, Val, and Ile. These inhibitors are likely to directly interact with the active site of the enzyme, causing the disruption of substrate binding [83]. Based on this work, a recent study reported an innovative virtual screening-based approach to select fragment-sized harmine-derived compounds and chelators of the catalytic magnesium of Mtb-SerB2. This work offered more insights into the necessary constituents for the interactions between the enzyme and inhibitors and provided interesting fragments for future fragment growth and drug discovery efforts [87]. The same research group recently published a study dissecting the complex oligomeric behavior of this protein. A combination of biochemical and biophysical experiments demonstrated that this enzyme exists in different oligomeric forms of different activity and has unique morpheein behavior, which is different from other mycobacteria orthologs. Apart from providing a deeper understanding of the mechanism of control of this protein, this study may also be the base for allosteric drug discovery and for future structure-based drug designs of inhibitors targeting oligomeric interfaces with the aim of increasing selectivity and avoiding concomitant human ortholog inhibition [88].

### 2.6. Inhibitors of Proline (Pro) Biosynthesis

**Figure 18:**
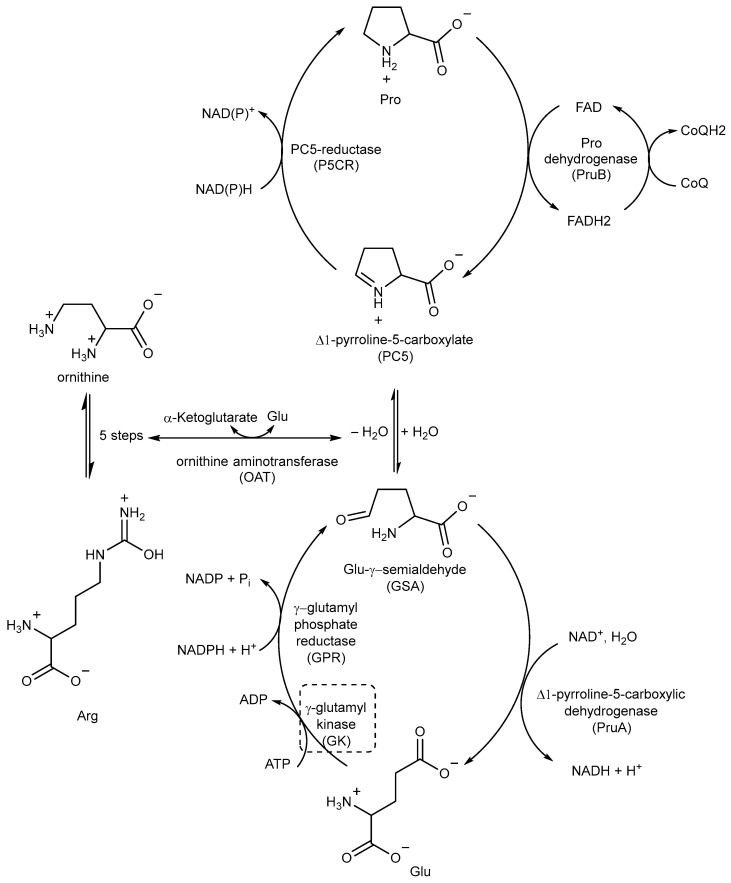
A schematic representation of the Pro metabolic pathway. The most relevant target is shown in a dotted box.

Pro biosynthesis starts with the phosphorylation of Glu to γ-glutamyl phosphate, which is catalyzed by the enzyme γ-glutamyl kinase (GK). γ-glutamyl phosphate reductase (GPR) then reduces γ-glutamyl phosphate to Glu-γ-semialdehyde (GSA), which undergoes cyclization to form Δ1-pyrroline-5-carboxylate (P5C). In the final step, P5C is further reduced to Pro by the enzyme P5C reductase (P5CR) using either NADH or NADPH as cofactors (Figure 18) [89,90].

P5C, the precursor of Pro, can also be obtained from ornithine through ornithine aminotransferase (OAT) (Figure 18). This important connection between Pro and Arg metabolism highlights the importance of Arg as an important source of nitrogen and carbon in *Mtb*, contributing to the overall metabolic pathways involved in nitrogen and carbon metabolism [41,89].

Pro catabolism is also a crucial factor in the ability of *Mtb* to persist within the host. The conversion of Pro to Glu is regulated by two specific enzymes: Pro dehydrogenase (PruB), which catalyzes Pro oxidation to Δ1-pyrroline-5-carboxylic acid (P5C) using a flavin cofactor, and Δ1-pyrroline-5-carboxylic dehydrogenase (PruA), which converts the tautomeric form of P5C (Glu-γ-semialdehyde) into Glu using NAD^+^ as a cofactor. These enzymatic reactions play key roles in the Pro metabolic pathway of *Mtb*, contribute to its ability to persist and thrive in the host environment, and protect it from methylglyoxal, a toxic and electrophilic metabolic intermediate [91,92]. However, even though studies could help in designing inhibitors, the potential of these enzymes as TB drug targets remains unexplored.

**Figure 19:**
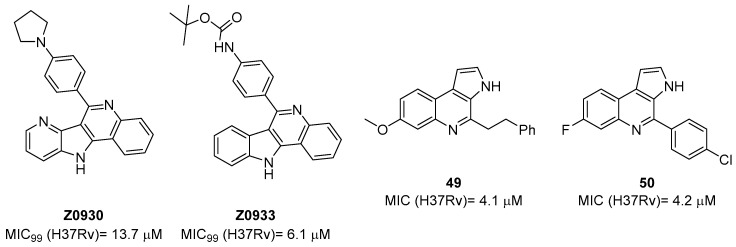
Chemical structures of **Z0930**, **Z0933**, **49**, and **50**.

The first study on GK, as a potential drug target, only appeared in 2019 while studying the mechanism of action of **Z0930** and **Z0933** (Figure 19), two compounds synthesized in the hit-to-lead optimization of a [3,2-c]quinoline scaffold, identified by the whole-cell screening of a diversity-oriented in-house library [93]. GK consists of two distinct domains: the main catalytic domain, AAK, which is responsible for binding and interacting with the substrate(s), and the PUA domain. Its catalytic activity can be regulated by the presence of *L*-Pro, which can act as a feedback inhibitor [93]. WGS of **Z0930**- and **Z0933**-resistant mutants revealed mutations within the *Rv2439c* gene, encoding for GK. Intriguingly, kinetic and biochemical studies demonstrated that these compounds enhance GK catalytic activity in a dose-dependent manner. Moreover, their activation kinetics do not follow a competitive pattern with ATP, indicating the existence of a separate allosteric regulatory site in GK, which is distinct from the ATP binding site [93]. Upregulated Pro production may eliminate *Mtb* both in vitro and in macrophages through the generation of ROS, disrupting cellular redox homeostasis and leading to cell death. A preliminary SAR analysis suggested that the heterocyclic framework is essential for GK activation. In particular, the quinoline core, which is shared by both compounds, is crucial for the binding of these compounds to GK [93]. This groundbreaking study not only discovered and unveiled the potential of a new drug target, advancing the knowledge and the importance of the Pro biosynthetic pathway, but also highlighted enzyme activation as a new opportunity in TB drug discovery. Inspired by this work, a more recent study identified two pyrroloquinolines, compounds **49** and **50** (Figure 19, with promising efficacies against *Mtb* (MIC = 4.1 and 4.2 μM, respectively)) [94]. Compound **50** binds to an allosteric pocket adjacent to the catalytic site responsible for L-Glu binding, whereas inhibitor **49** specifically interacts with a pocket in proximity to the ATP adenosine group recognition site. In silico studies predicted that compound **49** could hamper ATP binding by inducing long-distance conformational alterations in the ATP binding site. On the other hand, compound **50** does not influence ATP recognition and could cause conformational modifications in the *L*-Glu catalytic site, thereby preventing the anchoring of *L*-Glu which is necessary for catalysis. The discovery of these promising structural frameworks paves the way for the development of allosteric inhibitors targeting the GK enzyme and further supports the therapeutic potential of this biological target in the pursuit of novel anti-TB drugs [94].

### 2.7. Inhibitors of Asparagine (Asn) Metabolism

Ans and Asp are among the major sources of nitrogen for *Mtb*. The mycobacterium mainly exploits Ans from host tissues through the transporter AnsP1 and the paralogue AnsP2 (*Rv0346c*), which are involved in Asp and Ans uptake in the mycobacterial phagosome [9,95]. Even though AnsP2 is not responsible for virulence, *ansP2* expression is strongly induced in the lungs of TB patients, which could reflect the importance of this transporter. Ans catabolism supports acid stress resistance and intracellular survival. The only enzyme of the asparaginase family encoded by the *Mtb* genome is AnsA, which is secreted in the mycobacterial cytosol and outside the mycobacterial envelope and is responsible for the hydrolysis of Ans giving Asp and ammonia (NH_3_). NH_3_ is spontaneously protonated in the phagosomal lumen, reacting with protons transported by V-ATPase to form ammonium ions. This allows for phagosomal pH buffering and represents an essential strategy to resist acid stress both in vitro and inside macrophages [96,97]. Indeed, an *Mtb* mutant lacking the encoding gene *ansA* shows impaired nitrogen incorporation from Asn and has attenuated virulence in interferon gamma (IFN-γ)-activated macrophages and in mice [9]. Nonetheless, compared to other metabolic enzymes involved in nitrogen metabolism, those belonging to Ans metabolism are largely unexplored [95]. The only study highlighting the potential role of asparaginase as a potential drug target reports a three-dimensional structural model via the SWISS-MODEL server (http://swissmodel.expasy.org/) using *P. horikoshi* L.-asparaginase as a structure template (percentage of sequence identity equal to 27%). A comparison with asparaginase of other pathogens and the human one revealed a significant difference between them, suggesting that it could be a good and safe target. Potential inhibitors were selected from the Traditional Chinese Medicine (TCM) database, the ZINC database, and the FDA-approved drug database and predicted through a virtual screening against AnsA. Positive hits showed satisfactory activities against *M. smegmatis*, but the results have not yet been confirmed in *Mtb* [98].

### 2.8. Inhibitors of the Asp Metabolic Pathway

**Figure 20:**
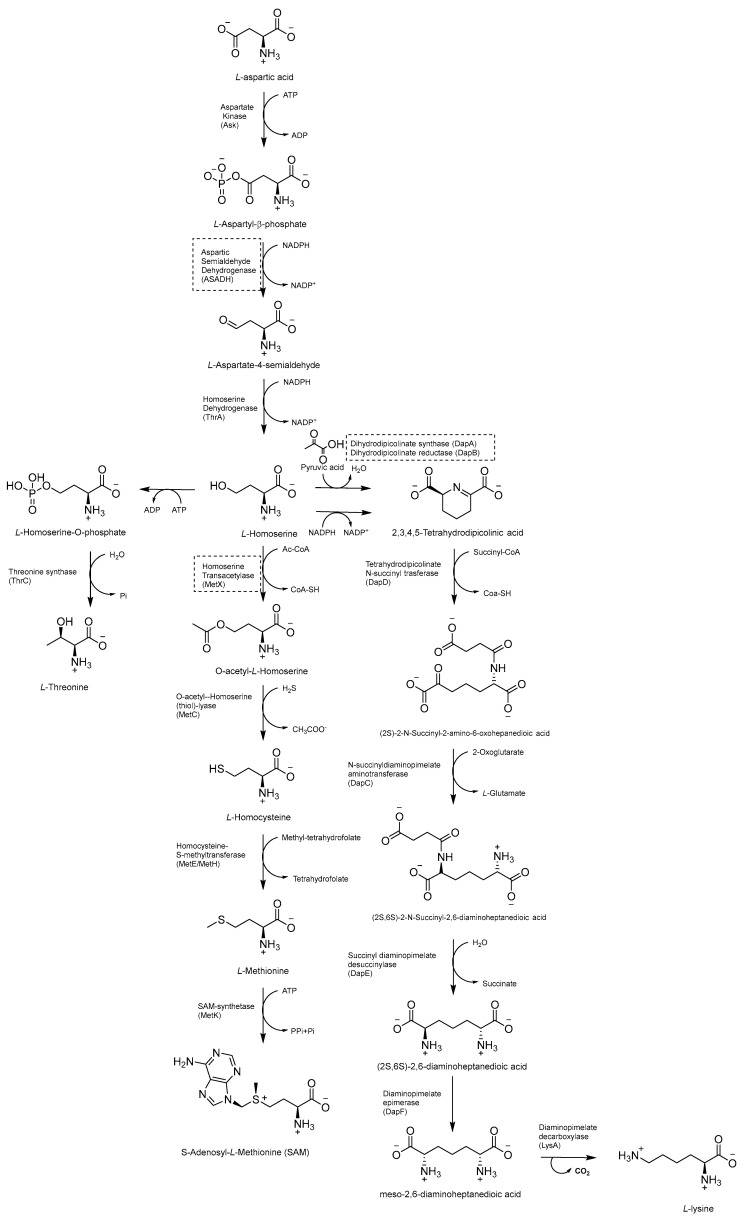
Schematic representation of Asp metabolic pathway.

The Asp metabolic pathway (Figure 20) exists in plants, fungi, archaea, and microbes, but not in mammals. It produces Met (Met), threonine (Thr), isoleucine (Ile), lysine (Lys), S-adenosyl-L-methionine (SAM), and diaminopimelate (DAP), a cell wall constituent that is important for the virulence of *Mtb* [99]. The disruption of the Asp pathway leads to a complex imbalance of metabolic fluxes, which is counteracted by *Mtb* through different compensatory mechanisms. Thr, homoserine, and Met auxotrophies exhibited a rapid cell death phenotype, confirming the vulnerability of this pathway and that both Met and Thr are required for *Mtb* survival. Thr starvation leads to an accumulation of Lys and homoserine metabolic intermediates. Indeed, Asp kinase (AK), the first enzyme of the pathway (refs. [10,11]) is controlled by Thr allosteric feedback, but not by Met or Lys. Instead of the ubiquitous Lys-AK feedback loop regulation, *Mtb* regulates Lys accumulation through two unique mechanisms, Lys degradation and export. Moreover, conditional knockdowns of *thrA* and *metX* revealed that these two branch point enzymes are required for late-stage infection. This demonstrates that the inability to scavenge Met, Thr, and homoserine from the host affects both the establishment and late-stage persistence of the infection, which is an essential feature for future drug discovery efforts. Therefore, due to its branched nature and multifaceted bactericidal mechanism, along with its importance in both acute and chronic infections, the Asp pathway offers a wide and still unexplored target space for anti-TB drug discovery [10].

#### 2.8.1. Aspartic Acid Semialdehyde Dehydrogenase (ASADH) Inhibitors

**Figure 21:**
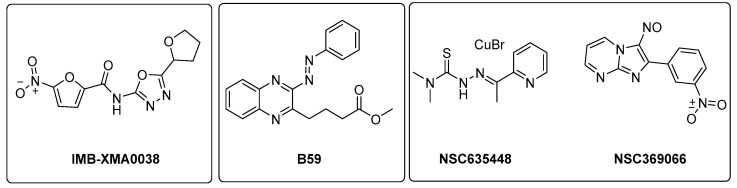
Chemical structures of **IMB-XMA0038, B59, NSC635448**, and **NSC369066**.

ASADH is a critical branch point for the biosynthesis of Lys, Thr, Met, and isoleucine [99]. Even though several inhibitors were identified through in silico approaches [100,101], the first study validating the efficacy of ASADH inhibitors in an experimental model only came out in 2021. Wang and coworkers identified **IMB-XMA0038** (Figure 21) through the HTS of 150,000 compounds using a surface plasmon resonance (SPR) assay as a secondary screening method for hit validation and a molecular docking analysis to predict interaction with the target [102]. The further characterization of in vitro **IMB-XMA0038** activity revealed that this compound is not only active against drug-sensitive strains (MIC = 1.7 µM), but also against clinical isolates (MICs = 1.7–3.4 µM) and dormant bacteria [103]. In addition, it showed a clear synergistic effect when combined with INH, RIF, bedaquiline (BDQ), moxifloxacin (MFX), and delamanid (DLM) because it is likely to improve drug penetration, which ultimately inhibits the synthesis of *Mtb* cell walls. The post-antibiotic effect (PAE) was also studied using *Mtb* H37Rv cells grown to the midlogarithmic phase (OD_600_ = 0.5). The PAE is an important pharmacodynamic indicator, resulting in a delayed resumption of bacterial growth following the removal of antibiotics from the cell culture. In this case, **IMB-XMA0038** acted in a dose-dependent manner, like INH. Finally, it reduced the bacterial load by 1.67 log_10_ in a murine model of acute *Mtb* H37Rv infection, confirming its potential as an anti-TB hit for further hit-to-lead optimization. Further studies are also needed to elucidate its mechanism of action and investigate its effects on cell wall integrity [103].

#### 2.8.2. Inhibitors of Lysine (Lys) Metabolic Branch

The Lys metabolic branch, also known as the diaminopimelate DAP pathway, is absent in mammals and is crucial for producing other important metabolites, such as dipicolinate and DAP, which are essential for the cross-linking of peptidoglycan polymers in bacterial cell wall synthesis [104,105]. It involves several enzymatic steps (Figure 20), starting from aldol condensation catalyzed by the enzyme dihydrodipicolinate synthase (DapA) to convert homoserine and pyruvate to dihydrodipicolinate. The latter is reduced by dihydrodipicolinate reductase (DapB) to tetrahydrodipicolinate, which, in turn, is converted to meso-DAP. Meso-DAP is then converted to Lys by diaminopimelate decarboxylase (LysA), a pyridoxal 5′-phosphate (PLP)-dependent enzyme [106] (Figure 20). The genes of this pathway are essential for *Mtb* growth, and some of them have already been investigated as targets for potential inhibitors [107,108,109,110]. The importance of dihydrodipicolinate reductase (DapB), encoded by *dapB*, has only been recently elucidated [104]. This enzyme uses NADH/NADPH as a cofactor to reduce dihydrodipicolinate to tetradihydrodipicolinate. It is composed of four identical subunits, each of them comprising two domains connected through a flexible region. The N-terminal domain binds the cofactor, while the C-terminal domain is responsible for substrate or inhibitor binding. An antisense *dapB* knockdown mutant strain exhibited growth defects and a reduced ability to infect macrophages, proving the importance of DapB in mycobacterial survival and virulence and its potential as a drug target. A virtual screening of 95 k molecules from an NCI database, followed by docking studies, identified 60 potential DapB inhibitors. The hit of this series was quinoxaline derivative **B59** (Figure 21), showing good enzymatic activity (32.9 µM), a reasonable MIC (59.8 µM), and negligible cytotoxicity against three different cell lines. This offered a proof of concept that DapB is a potential drug target and that **B59** is worth investigating with further hit-to-lead optimization studies to enhance its antimycobacterial activity and improve its cell permeability and drug-like properties [104].

#### 2.8.3. Inhibitors of Met Metabolic Branch

The essential branch point of the Asp pathway for Met biosynthesis is the conversion of homoserine to O-acetyl-L-homoserine (OAHS) catalyzed by the enzyme homoserine transacetylase (MetX) [10] (Figure 20). MetX exploits acetyl-CoA as a cofactor and has a conserved catalytic triad comprising Ser157, His350, and Asp320 and Ser157 as a nucleophile. The catalyzed reaction follows a ping-pong mechanism: once activated, Ser157 reacts with acetyl-CoA, producing the acetyl-enzyme intermediate and CoA. Following the dissociation of CoA, it binds *L*-homoserine, and then the γ hydroxyl of L-homoserine attacks the acetyl–enzyme intermediate complex, generating an OAHS product and free enzyme [111].

Met is an important factor for translational initiation and is the precursor of SAM, a cofactor involved in biochemical reactions and regulatory mechanisms. Both Met and SAM are involved in essential roles in *Mtb*, such as the control of the eukaryotic cell cycle, autophagy, and differentiation of human pluripotent stem cells. Moreover, SAM is a cofactor for one-carbon metabolism, and it is responsible for the methylation of DNA, RNA, proteins, and lipids by SAM-dependent methyltransferases [11]. The deletion of MetX (encoded by the *Rv3341* gene) generates auxotrophic mutants that are unable to establish a productive infection both in immunocompetent and immunocompromised mice. Compared to other amino acids, this auxotrophy has a strong bactericidal effect, which rapidly leads to mycobacterial death. This indicates that *Mtb* is completely dependent on this pathway for its survival and suggests a multitarget cell death mechanism. Consistent with their composite biological roles, the blockade of Met and SAM production causes pleiotropic effects, including the inhibition of several methyl-transferase-dependent processes, a stalling of translation initiation, the toxic accumulation of Lys, and a pervasive metabolic shutdown. Therefore, the Met metabolic branch provides an excellent drug target space. Recent structural studies aiming to elucidate the druggability of MetX have revealed the existence of druggable sites, confirming that this is an excellent candidate for structure-based small-molecule drug discovery [111]. The first HTS assay for identifying MetX inhibitors was developed in 2022 by Chaudhary and coworkers, who screened a library of 2334 compounds of the NCI-DTP library and identified two primary hits (**NSC635448** and **NSC369066**) that are active against MetX in a dose dependent manner with MIC values of 3.125 µM and 6.25 µM, respectively (Figure 21). Docking studies revealed that compound **NSC635448** forms a hydrogen bond with Tr61 residue, while **NSC369066** is involved in the hydrogen bond with Leu60. **NSC369066** seems to be the best compound, also fitting in the binding pocket for interactions with Arg227, Lys272, and Arg276 residues, which are located near the active site. **NSC369066** was the most potent, with killing levels comparable to INH, which were partially restored after Met supplementation. Unfortunately, this first-in-class MetX inhibitor suffers from high cytotoxicity (5 µM) and multitarget killing activity, and further effort is required to improve its antimycobacterial activity and its SI before considering it as a promising candidate for further hit-to-lead optimization [112].

### 2.9. Inhibitors of Cysteine (Cys) Biosynthesis

**Figure 22:**
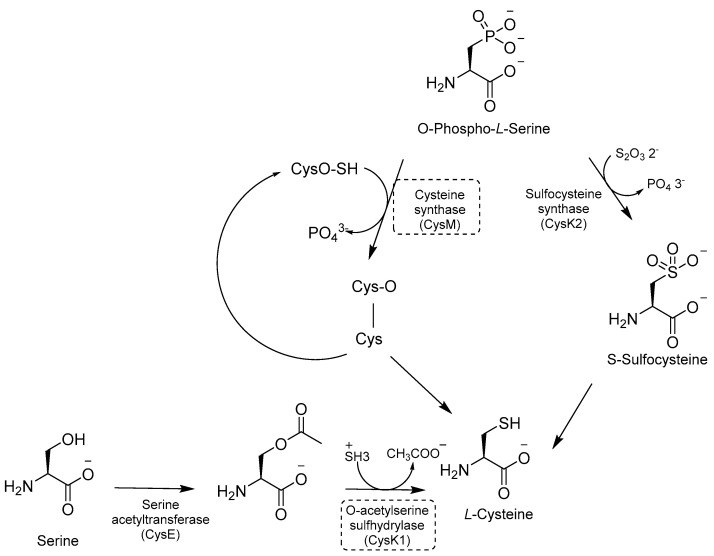
A schematic representation of Cys biosynthesis. The most relevant targets are shown in dotted boxes.

Cys is required for mycobacterial growth and is involved in mycothiol biosynthesis, which is necessary to maintain redox homeostasis in dormant models. Cys contributes to the repair of the iron–sulfur center containing proteins, which is damaged by ROS and reactive nitrogen intermediate (RNI) species. There are three distinct pathways for de novo Cys biosynthesis. Each of them is characterized by a different PLP-dependent Cys synthase: (i) CysK1, a bona fide PLP-dependent O-acetylserine sulfhydrylase encoded by the gene *rv2334*, which converts O-acetylserine and sulfide to Cys; (ii) CysK2, a sulfocysteine synthase encoded by the gene *rv0848*, which converts O-Phospho-*L*-Ser into S-sulfocysteine and then into *L*-Cys; and (iii) CysM, a unique sulfhydrylase for *Mtb* encoded by the gene *rv1336* [113] (Figure 22).

Inhibitors developed in the past decade have provided valuable tools to investigate the role of the Cys pathway in *Mtb* (Table 3) [114,115,116,117]. However, in vivo efficacies remain to be tested, along with the need for clear target validation. Therefore, none of them have further progressed, and research in this field has been stalled in the past five years. Moreover, the existence of three different pathways is intricated, and further gene knock-out and animal models are needed to demonstrate their essentiality for *Mtb* survival [113].

Besides the de novo biosynthetic pathway, Cys can also be produced by a less known and poorly characterized reverse transsulfuration pathway, which is the only source of glutathione and Cys in eukaryotes. Cystathionine β-synthase (MtbCbs) has recently been characterized and appears to be an important regulator of sulfur metabolism in *Mtb*, but its druggability has not yet been validated [118].

**Table 3:** Chemical structures, SAR plan, and most active inhibitors of Cys biosynthesis.

Target	Hit/Scaffold	SAR Plan from Hit	Most Advanced Analogue	Refs.
CysK1	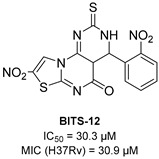	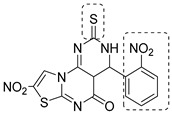	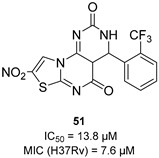	[114]
CysK1	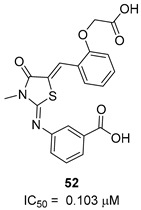	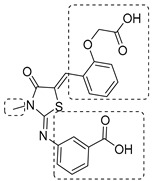	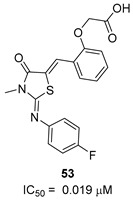	[115]
CysM	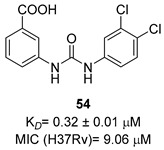	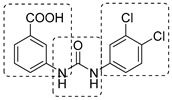	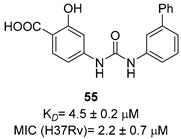	[116,117]

### 2.10. Inhibitors of Histidine (His) Biosynthesis

Besides its proteogenic and catalytic functions, His is also an important regulator of several cellular processes, such as cellular pH maintenance and metal chelation. While bacteria and plants possess enzymes for de novo biosynthesis, it is an essential amino acid for humans. Even though enzymes of this biosynthetic pathway have been explored for decades as potential targets for TB treatment [119], the importance of His in an in vivo setting was only elucidated recently [120]. His is one of the most abundant amino acids in the host intracellular milieu. Nonetheless, His auxotrophs fail to infect immunocompetent mice, as His restriction is a host adaptive immunity mechanism to contain *Mtb* infection. When *Mtb* infects macrophages, a CD4 T-cell-mediated mechanism induces an enhanced production of IFN-γ, which activates downstream signaling to upregulate His ammonia-lyase (HAL) and His decarboxylase (HDC), two host catabolizing enzymes. Similar to Trp, this mechanism reduces His availability to *Mtb*, which counteracts the immune response by activating the de novo His biosynthetic pathway. His biosynthesis is then an immune evasion strategy and is vital for *Mtb* to persist in the host microenvironment. Therefore, inhibitors of this pathway can synergize with the host in clearing the infection and can lead to rapid bacterial clearance.

**Figure 23:**
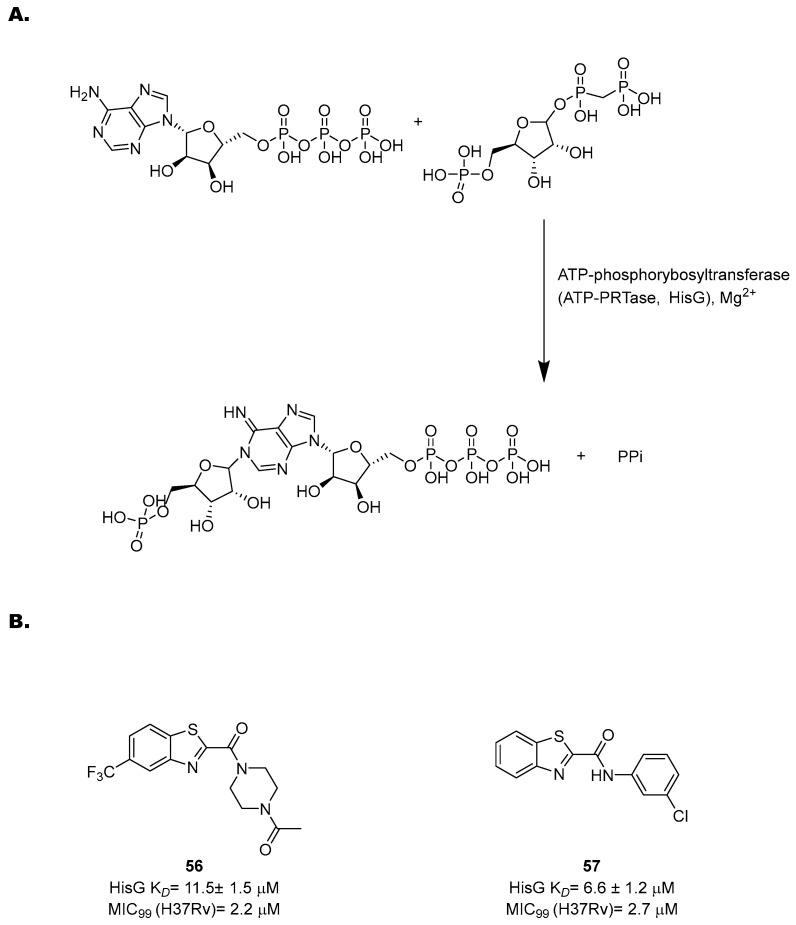
(**A**) A schematic representation of the first step of His biosynthesis, catalyzed by HisG; (**B**) the chemical structures and antimycobacterial activities of compounds **56** and **57**.

The His pathway starts from phosphorybosylpyrophosphate (PRPP), which is converted to His through ten enzymatic reactions [119]. Many enzymes of this pathway have been biochemically and structurally characterized to guide the rational development of specific inhibitors based on a structure-based approach [121,122,123,124,125,126]. However, only few of them were validated through target-specific inhibitors. One of the most well-characterized targets is an ATP-phosphorybosyltransferase (ATP-PRTase, HisG), which catalyzes Mg^2+^-dependent nucleophilic substitution of PRPP using ATP to produce phosphoribosyl-ATP and inorganic pyrophosphate (Figure 23A).

With the His pathway being metabolically expensive (10 enzymatic reactions and 41 ATP molecules consumed), the activity of HisG is allosterically regulated by His through negative feedback [122,127]. Transposon insertion experiments [128] and knockout studies [129] showed that it is essential for in vivo growth, highlighting its potential as a drug target. In 2008, a virtual screening of more than 500,000 molecules identified diverse potential inhibitors [127]. Interestingly, several of the hits contain a nitrobenzothiazole fragment, opening the way to new possibilities for drug design and hit optimization against this target. Inspired by these findings, Dhameliya and coworkers have explored the potential of this scaffold in the past decade [130,131]. Hit optimization studies allowed for the identification of two new chemotypes, benzo[*d*]thiazole-2-carboxamides and benzo[*d*]thiazole-2-carbanilides, which are devoid of the potential mutagenicity of the nitro group and match with the topological features of PR-ATP, the natural ligand of PRPP transferase. An extensive SAR study led to **56** and **57**, the most potent HisG inhibitors reported to date, with EC_50_ values of 20 ± 2.2 and 14 ± 1.8 µM, respectively, good antimycobacterial activities (2.2 and 2.7 µM, respectively), and non-cytotoxic behavior (Figure 23B). A His complementation assay confirmed that these compounds acted on His biosynthesis. Biochemical and biophysical studies revealed competitive inhibition towards ATP and also revealed that they induced a significant change in the HisG secondary structure. These data were corroborated by docking and molecular dynamics simulations, suggesting that they form a stable complex with the target and bind to the catalytic cleft at the interface between Domain I and Domain II.

Looking at the future, two interesting targets of recent characterization are imidazoleglycerolphosphate dehydratase (IGPD) and histidinol phosphate phosphatase (HolPase), catalyzing the sixth and the eight steps of the pathway, respectively [124,126].

### 2.11. Inhibitors of Ala Biosynthesis

Ala is a substrate for protein synthesis and an important component for the synthesis of cell wall peptidoglycan. It is synthesized by Ala dehydrogenase (Ald), a NADH-dependent enzyme which catalyzes the reductive amination of pyruvate and its reverse reaction [13]. While *ald* knockout strains are not auxotroph for Ala [12], the absence of Ald affects mycobacterial growth under anaerobic conditions [13]. This suggests that *Mtb* could possess an alternative Ala biosynthetic pathway and that Ald could be involved in mycobacterial survival under oxygen-limiting conditions [13,132,133]. The most important role of Ald is the regulation of redox homeostasis. Hypoxic and starvation conditions encountered by mycobacteria in the granuloma, as well as an altered function of the respiratory chain, cause a shift of the NAD^+^/NADH balance toward a reduced state, which promotes the Ald-mediated pyruvate conversion to Ala and concomitant NADH oxidation, increasing the Ala cellular levels [13]. Moreover, Ald activity is regulated by Ala availability, which, under anaerobic conditions, strongly upregulates *ald*. Differently from other biosynthetic pathways, the main aim of the application of Ald inhibitors is to disrupt the redox balance in nonreplicating mycobacteria rather than to target Ala biosynthesis to induce starvation. The availability of its crystal structure served as the structural framework for many virtual screenings against this target [134,135]. The most recent study identified a series of adenosine-based inhibitors using a versatile label-free assay [136]. A first round of screening identified an N6-methyladenosine inhibitor, which occupies the NAD binding site of Ald. This inhibitor was co-crystallized and optimized through a rational design based on the crystal structure. Since the N6-methyl moiety extends and interacts with a hydrophobic groove structure, a series of N6-alkyl bulkier analogues was synthesized to evaluate hydrophobicity contributions and increase affinity by extending hydrophobic interactions. These structural changes substantially improved the affinity for the target, increasing the affinity by a factor of ten (N-6-isobutyl derivative K*_i_*= 80 µM). This study describes a very versatile screening platform, providing an efficient drug discovery strategy which could serve to identify several hits against this target. However, it is limited by the absence of activity confirmation in *Mtb*.

## 3. Conclusions

Although it is responsible for a preventable and curable disease, *Mtb* remains among the world’s top infectious killers, causing over one million deaths annually. Significant concern arises from the emergence of MDR and XDR strains, fueled by the length of anti-TB treatment and HIV comorbidity. Indeed, the rise of drug resistance has significantly increased treatment failure rates and the need for long and far more toxic and costly therapies [137]. To shorten treatment duration, an ideal anti-TB drug should be amenable to oral administration, safe in association, less toxic, and more effective and have an innovative mechanism of action. In recent decades, a renewed interest in TB drug discovery and the combined efforts of academia and industry have significantly fueled the TB drug pipeline, providing a positive perspective of the future of TB drug discovery. Even though this pipeline contains novel chemical scaffolds and a variety of targets, there are still some redundancies, and drugs acting with novel mechanisms of action remain unrepresented. Until recently, *Mtb* metabolism has been seldom studied from a drug discovery perspective due to the belief that *Mtb* can scavenge amino acids from the host and subsequently reverse the activity. More recently, several studies with amino acid auxotrophs [5] have reconsidered this hypothesis, proving that subverting metabolic restrictions is an essential mechanism to escape host immune surveillance and that unbalanced metabolic fluxes are bactericidal during different stages of infection. Therefore, altering amino acid metabolism and host responses critical for amino acid starvation could provide new avenues for TB drug discovery [6]. However, not all metabolic pathways are suitable for targeting, as some of them are produced by multiple enzymes. Moreover, information regarding amino acid transporters is limited, which hampers our understanding of competition mechanisms and the overall link between host and mycobacteria metabolism [138]. Therefore, it is crucial to understand which pathways are essential, which enzymes are the most important, and whether these enzymes are druggable [5]. In the past five years, the multifaceted bactericidal mechanism of amino acid starvation has gained considerable attention, and a better understanding of amino acid biosynthetic pathways has paved the way for target-based screenings against defined protein targets of the pathways. Many inhibitors of key enzymes of several amino acid biosynthetic pathways have been discovered, proving their chemical vulnerability and providing optimism for future advances [95]. While several studies have reported nanomolar enzymatic inhibitions, there is still a lack of cell and animal experiments. Many studies are still at the level of in silico prediction or biochemical experiments, with only a few demonstrating documented activity against whole-cell *Mtb*. For many of these potential inhibitors, crossing the complex *Mtb* cell envelope and avoiding efflux expulsion to engage their targets could be extremely challenging [55]. As frequently occurs with novel pathways, research has focused more on validating targets and discovering new chemical scaffolds despite optimizing existing hits. Yet many hits have been discovered over the past five years, most of them still suffer from low bactericidal activity and a lack of in vivo validation, and extensive improvements in their drug-like properties are required. Among these, Trp inhibitors stand out as the most advanced and have significantly contributed to demonstrating the feasibility of this approach for future TB drug discovery. Future efforts should be directed at prioritizing the chemical optimization of these hits to enrich the TB drug pipeline with high-quality leads.
